# Evaluation of antifungal activity of *Lysinibacillus fusiformis* as a biocontrol agent against *Rhizoctonia solani* in soybean

**DOI:** 10.3389/fpls.2026.1902846

**Published:** 2026-08-10

**Authors:** Faisal Ay Alzahrani, Mehran Ullah, Mohsen M. Elsharkawy

**Affiliations:** 1 Department of Chemistry, College of Sciences and Arts, King Abdulaziz University, Rabigh, Saudi Arabia; 2 Department of Agriculture, Faculty of Environmental Sciences, King AbdulAziz University, Jeddah, Saudi Arabia; 3 Department of Agricultural Botany, Faculty of Agriculture, Kafrelsheikh University, Kafr Elsheikh, Egypt

**Keywords:** biological control, crop parameters, induced systemic resistance, *Rhizoctonia solani*, rhizosphere bacteria, soybean

## Abstract

**Introduction:**

*Rhizoctonia* root rot has a wide host range and no resistant soybean cultivars exist, making it a serious problem.

**Methods:**

The antifungal activity of 65 isolates of rhizobacteria against *R. solani* was evaluated.

**Results and Discussion:**

The pathogen in the in vitro experiment was inhibited by nine rhizobacterial endophytes. The most effective rhizobacterial isolate was genus-level verified using 16S rDNA partial sequence analysis. The impact of the biocontrol isolate was investigated on *R. solani* infection in both greenhouse and field conditions. The rhizobacterial isolate considerably decreased the percentage of pre- and post-emergence damping-off and enhanced the plant survival compared to the untreated control grown in soil infected with *R. solani*. *Lysinibacillus fusiformis* treatment resulted in significantly stimulated antioxidant enzymes and total phenols. Transcript levels of the studied defense-related genes were considerably elevated after *L. fusiformis* treatment, according to quantitative RT-PCR data. Treatment of *L. fusiformis* in challenged plants achieved the highest levels of PR-1, PR-2, PR-10, and PR-12. The field experiments revealed that the biocontrol treatment method significantly reduced *Rhizoctonia* root rot incidence, stimulated plant growth, and increased yield. This research has uncovered a promising biocontrol bacterial isolate that might be used as a novel bioagent for controlling *R. solani*. After further toxicological and ecotoxicological evaluation, formulation development, and multi-location field trials, *L. fusiformis* may represent a promising candidate for development as a biocontrol agent.

## 1 Introduction

Soybean is one of the world’s most important oilseed and protein crops. Recently, soybeans have become a major player in the global protein market. Fungal infections are one of the biotic stressors that lead to yield loss and significant social and economic issues in the world. Many infectious diseases affecting agricultural plants are caused by oomycetes and phytopathogenic fungi (Amin et al., 2026). Soybean yield is reduced due to root rot, which is caused by *R. solani* (Paliwal et al., 2023). Chemical pesticides are often used to manage this disease, but they may lead to pollution and the development of pesticide resistance if used over an extended period of time (Ludueña et al., 2012). Organic farming practices limit the usage of several pesticides. Consequently, the interest in developing biological control to avoid phytopathogen infection has gradually increased (Elsharkawy et al., 2022). Biological control provides an effective and eco-friendly alternative to conventional pest management by using beneficial microbes.

Plants have developed a wide array of defense mechanisms to protect themselves from harmful infections (Elsharkawy et al., 2022). Defense mechanisms, such as cell wall thickening, active oxygen bursts, and accumulation of defense enzymes are induced by beneficial bacteria, which in turn suppress phytopathogenic fungi (Lu et al., 2017). Plants may develop systemic immunity via mechanisms such as ISR (induced systemic resistance). PGPR (Plant growth-promoting rhizobacteria) and other beneficial microbes activate ISR to modulate signaling pathways reliant on JA (jasmonic acid) and ET (ethylene) to boost plant immunity (Pieterse et al., 2009). A novel biological strategy for disease management in plants is the induction of defense mechanisms. Environmentally friendly strategies of crop management are promising especially using microorganisms that may directly antagonize fungal diseases by competing for key nutrients or by developing biofungicides (Tóth and Stacey, 2015). One potential source of plant health stimulants is the existence of mutualistic plant-microbe relationships, which are known to offer physiological and molecular advantages (Carvalhais et al., 2013). The nutrient cycling actions of PGPR improve the availability of important nutrients and stimulate plant development (Wahyudi et al., 2011). Additionally, certain bacteria may boost plant resistance against plant diseases. The host may undergo physiological changes and defensive induction in response to these PGPR, which may have systemic impacts on pathogenic populations both above and below ground levels (Pineda et al., 2013). The ability of several *Bacillus* species to induce systemic resistance in plants against harmful fungi is a topic of active research (Maslennikova et al., 2023). Jain et al. (2017) reported that the expression of defense-related genes and the systemic activity of enzymes such as POD (peroxidase), SOD (superoxide dismutase), PPO (polyphenol oxidase), and PAL (phenylalanine ammonia lyase) were increased in soybean by the presence of *Bacillus* sp. The upregulated transcripts of defense gene expressions by treatment with rhizobacteria may also be involved in protecting plants against pathogens (Elsharkawy et al., 2022). The viability and adaptability of the PGPR inoculum with the local microbes are strain-dependent and greatly affected by the application procedures which sometimes lead to inconsistent results when tested in the field (Pellegrini et al., 2020). Therefore, potential application methods of PGPR should be utilized (El-Gendi et al., 2022). The biotechnological potential of *L. fusiformis* strains has attracted attention in recent years. These strains are particularly good in releasing important chemicals and enzymes, such as peptidases and esterases (Jabeur et al., 2020). Additionally, *L. fusiformis* has gained a lot of attention due to its possible anti-fungal activity against plant infections under laboratory conditions (Pudova et al., 2018).

The activities of defense enzymes in the soybean cellular defense response should be described in an attempt to uncover the underlying mechanisms of soybean systemic resistance caused by the rhizobacterial isolate. Additionally, the transcription levels of genes associated with defense and pathogenesis-related proteins should be assessed. The antifungal activity of the *L. fusiformis* was assessed under greenhouse and field conditions.

## 2 Materials and methods

### 2.1 Soybean seeds and microbial cultures

*Rhizoctonia solani*, the pathogenic fungus, was obtained from the Plant Pathology Lab., Kafrelsheikh University, Egypt. *R. solani* was cultured at 28 °C for three to four days on a PDA plate. The rhizosphere of *R. solani*-infected soybean in Kafr Elsheikh, Egypt, is the original source of the biocontrol isolate. The isolate was characterized using full-length 16S rDNA sequencing and officially submitted to the NCBI GenBank (Elsharkawy et al., 2022). The biocontrol isolate was propagated in a growth medium containing NB (Nutrient Broth) at 37 °C and 180 rpm for 24 h in a shaker until the concentration reached 10^8^ cfu/ml (Huang et al., 2017). Soybean seeds (cv. Giza 111) were obtained from the Field Crops Research Institute, Agriculture Research Center, Egypt.

### 2.2 Antifungal activity of the rhizobacterial isolate *in vitro*

*In vitro* antifungal efficacy of the biocontrol isolate against *R. solani* was assessed. A dual-culture plate approach was used. Mycelial culture disks of *R. solani* cultures (5-day-old, with a diameter of 6 mm) were moved to one side of the Petri dish that contained PDA. The bacterial culture was smeared just below the plate’s center on the other side of the mycelia disk in order to test for antifungal activity. It was then maintained at 25 ± 2 °C in the incubator until *R. solani* attained full development in the control plate.

### 2.3 Microscopic examination

The impact of the rhizosphere bacteria on the development of *R. solani* was examined using SEM (scanning electron microscopy). Fixation and dehydration procedures were used to prepare the samples (Elsharkawy and El-Khateeb, 2019). The samples were left in a 0.1M phosphate buffer with 3% glutaraldehyde for 3.5 h at 24 °C. The samples received three rounds of washing with SDW (sterile distilled water). Samples were washed one more time after being post-fixed in osmium tetroxide for 2 h at 24 °C. Drying was performed at CPD (critical point) in ethanol concentrations. A sputter coating and metal coating were applied to the specimens. The samples were investigated using a TESLA BS-300 field emission electron microscope in Faculty of Agriculture, Mansoura University, Egypt.

### 2.4 *Rhizoctonia solani* inoculum preparation

*R. solani* cultures, that were seven-day-old, were used to produce fungal inoculum (Germain et al., 2021). Five hundred ml flasks containing PDB (potato dextrose broth) medium were used to inoculate *R. solani* disks. The mixture was then incubated at 24 ± 2 °C for 7 d using a rotary shaker set at 100 rpm. After harvesting the mycelia, they were homogenized in SDW and removed from the nutritional medium using No. 1 Whatman filter paper. Soil infestation was achieved by preparing an inoculum solution of *R. solani* mycelium with a final concentration of 2.2 mg dry weight/ml using sterile Milli-Q water.

### 2.5 Biopriming of soybean seeds

Seeds were “microbiolized” in accordance with Sharma et al. (2003). Soybean seeds were treated with a 24-hour-grown culture of the bacteria. After being exposed to a solution of HgCl_2_ (0.1%) and ethanol (70%) for 3 min, the seeds of soybean were immersed in Milli-Q water for multiple washings. The bacterial cultures that were cultivated overnight were spun in a centrifuge (at 10, 000 rpm for 20 min). The resulting pellets were then rinsed with 10 mM phosphate buffer saline. The OD540 was adjusted to 0.2 (10^8^ CFU ml^-1^) by resuspending the pellets in PBS containing 0.1% carboxymethylcellulose (CMC). The seeds were air-dried in a laminar hood after being surface-sterilized and then put in PBS with 0.1% CMC for 1 h. The experimental settings were kept sterile the whole time. The control group consisted of seeds that were submerged in autoclaved, non-microbiolized Milli-Q water.

### 2.6 Greenhouse experiment

Plastic pots (diameter 28 cm) were filled with disinfested soil (sandy clay, 1:2 v/v). Infested soil was watered for a week every 2 days before planting to encourage fungal development. Greenhouse conditions are temperature of 26 ± 2 °C, 60% relative humidity and 16:8 h light: dark photo period. Treatments were *R. solani* challenged plants, *L. fusiformis* treated and *R. solani* challenged plants, *L. fusiformis* treatment alone, and healthy plants. Each pot was grown with six pre-treated soybean seeds, and the pots received direct irrigation. Each treatment was evaluated using fifteen identical pots. After 15 days, the percentage of pre-emergence damping-off was calculated. After 30 days, the percentage of post-emergence damping-off was calculated. After 60 days, the percentage of infected plants was calculated (Nawar, 2007).

### 2.7 Assessment of growth parameters

Careful uprooting of plants occurred 60 days following sowing to avoid damaging root tissues. After being rinsed with Milli-Q water, five plants were randomly selected from each treatment replication. Shoot length and root and shoot dry weight were among growth metrics examined.

### 2.8 Assessment of chlorophyll

Methods for estimating chlorophyll were developed in accordance with those of Hiscox and Israelstam (1979). After removing and cutting the leaves into tiny pieces, 50 mg of leaf tissue was mixed with 4 ml of DMSO (dimethylsulfoxide). After incubating the tubes (that contain the leaf pieces and DMSO at 65 °C for 3 h), the extract was diluted with DMSO until reaching a volume of 5 ml. Then, the A^645^ and A^663^ were measured using DMSO as a control.

### 2.9 Antioxidant enzymatic activities and total phenol estimation

The application of rhizosphere bacteria was utilized to elicit defensive enzymes in soybean plants. The experimental design utilized both treated and non-treated plants. The enzyme activity (phenylalanine ammonia-lyase, lipoxygenase, peroxidase, and polyphenol oxidase) in various treatments was examined in soybean leaf and root extracts. Enzymatic assays were performed on plant samples taken after challenge inoculation to monitor changes in defensive enzyme activity. Samples of plants were taken from 10-day-old seedlings.

Quantification of phenol was carried out in accordance with McDonald et al. (2001). Homogenized leaf and root tissue samples (1 g) were mixed with 10 ml of 80% methanol and stirred for 15 min at 70 °C. A mixture of folin-Ciocalteau reagent (5 ml, 1:10 diluted with distilled water) and 4 ml of aqueous sodium carbonate (1 M) was prepared by adding 1 ml of the methanolic extract. Spectrophotometer readings taken at 765 nm indicated the generated blue color’s absorption. The standard was catechol.

PAL activity was determined using a refined technique developed by Mori et al. (2001). The process relies on the conversion of cinnamic acid from L-phenylalanine. The PAL activity was calculated as µmol of cinnamic acid min^-1^ mg protein^-1^ and was determined at 290 nm.

A procedure similar to that of Choudhary (2011) was used to ascertain POD activity. Guaiacol was used as a substrate to observe the absorbance at 436 nm. POD activity was reported as μmol product min^-1^ mg protein^-1^.

LOX activity was determined using the technique of Jain et al. (2014) based on the rise in absorbance at 234 nm due to the conjugated double bond system present in the hydroperoxide formed from linoleic acid.

PPO activity was assayed using a modification of the technique of Cavalcanti et al. (2007). Catechol was converted to o-quinone at wavelength of 410 nm.

### 2.10 Expression analysis of defense-related genes in soybean plants

To investigate the effects of *R. solani* infection and rhizobacteria inoculation on genes associated with plant defense, total RNA was isolated from the roots and leaves of soybean plants independently (Gao et al., 2012). Six-day-old soybean seedlings were used in this study. The leaves were quickly immersed in liquid nitrogen after being rinsed with tap water. The manufacturer-recommended protocol for the Plant RNA Mini Kit (Omega, GA, USA) was followed for the extraction and isolation of total RNA. The manufacturer’s instructions (Promega, Madison, USA) were followed for the synthesis of first-strand cDNA. An endogenous control gene, *EF1-α*, found in soybeans, was used for sample normalization. A set of four genes associated with plant defense was chosen for qRT-PCR (quantitative real-time PCR) analysis to confirm their expression levels. The primer sequences of soybean defense genes that were investigated are detailed in **Table 1**. The comparative 2^−ΔΔCt^ method technique was used to determine relative expression levels, using threshold values obtained from the ABI PRISM-7500 Software Tool (Livak and Schmittgen, 2001).

**Table 1:** The nucleotide sequences of the primers utilized in this study.

Primer	Forward	Reverse
*PR-1*	TGATGTTGCCTACGCTCAAG	AAGCAGCAACCGTATCATCC
*PR-2*	GTCTCCTTCGGTGGTAGTG	ACCCTCCTCCTGCTTTCTC
*PR-10*	GCCCAGGAACCATCAAGAAG	CGCTGTAGCTGTATCCCAAG
*PR-12*	CATGGACAAGGCACGATTTGG	AACCGATGGCTCTTTGACTCAC
*EF1-α*	TGCAAAGGAGGCTGCTAACT	CAGCATCACCGTTCTTCAAA

### 2.11 Field experiment evaluation

Experiments were carried out across two seasons (2024 and 2025) in Kafr Elsheikh, Egypt. A control group which did not receive any treatment, was also utilized for the evaluation. The fungicide Thiophanate-Methyl (Topsin M 70% W.P., 3 g fungicide kg^-1^ seeds) was used as a positive control seed treatment. After being treated, the seeds were spread out and dried as described previously. A randomized block design (RBD) was used with three treatments and six replications to instantly plant the treated seeds after drying. The dimensions of each gross plot were 6 rows, 3 meters long, with 45 cm between rows and 10 cm inside each row. The recommended and suggested package of procedures was followed for the nutrients and pest management.

### 2.12 Statistical analysis

The data were analyzed using ANOVA (analysis of variance) with a significance level of *p<0.05*, and the means were analyzed using Duncan’s multiple range test in IBM SPSS Statistics v. 22 (IBM Corp., Armonk, NY, USA). All of the findings were documented in triplicate.

## 3 Results

### 3.1 Rhizobacterial identification

**Figure 1:**
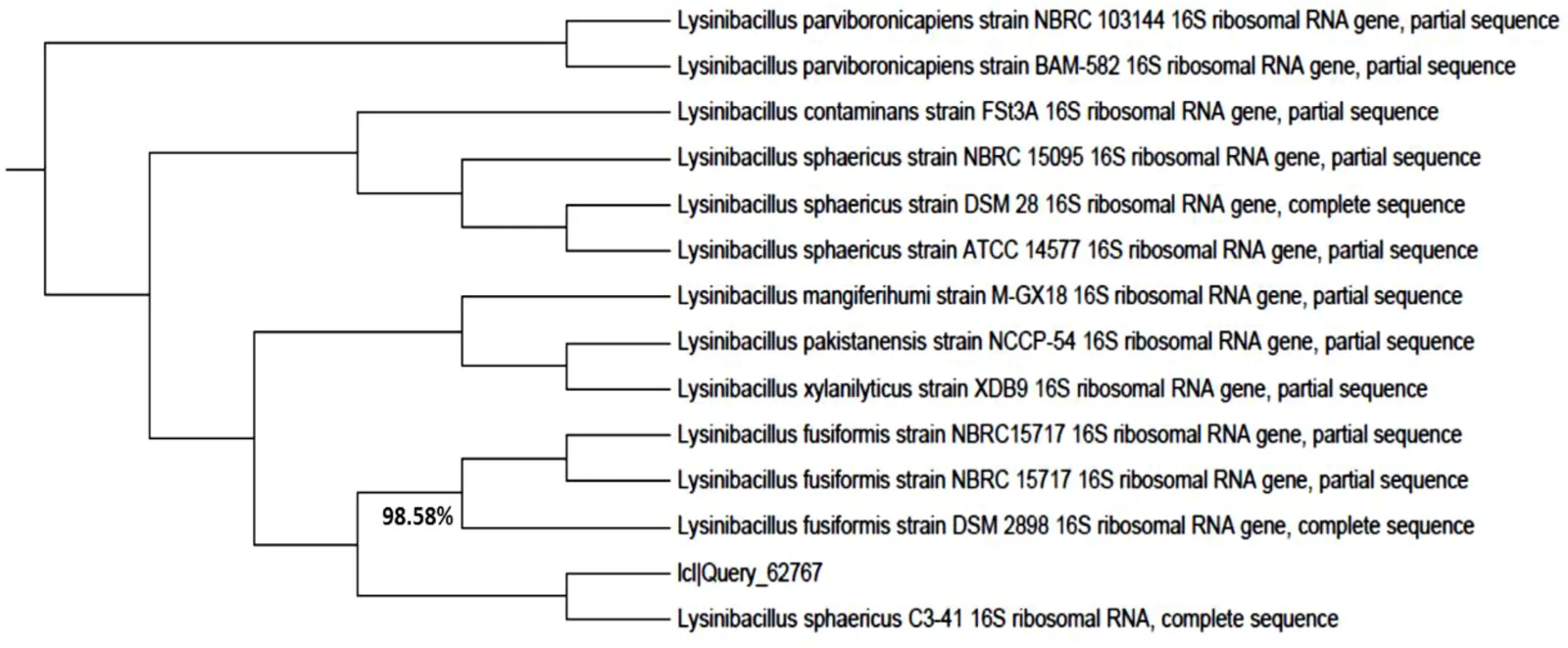
A phylogenetic tree, constructed using the MEGA12 program, demonstrated the connection between the identified *L. fusiformis* (accession number OP862866) and other *Lysinibacillus* isolates found in GenBank. Bootstrap values are based on 1,000 replicates.

The isolate that displayed the greatest antifungal activity was selected for identification and future usage. Molecular investigation of the GenBank sequence database indicated a 98.58% homology match with *Lysinibacillus fusiformis* strain DSM 2898, suggesting a close link between the two species. *L. fusiformis* 16S rRNA sequence of strain Elsharkawy (accession number OP862866) is deposited in GenBank. The phylogenetic tree was created, showing that *L. fusiformis* strain Elsharkawy is closely related to other strains of *L. fusiformis*, especially strain DSM 2898 (**Figure 1**).

### 3.2 Antifungal activity of rhizosphere bacterial isolate

The growth of *R. solani* is highly inhibited by *L. fusiformis*, suggesting that it could be a powerful antifungal agent in the environment.

**Figure 2:**
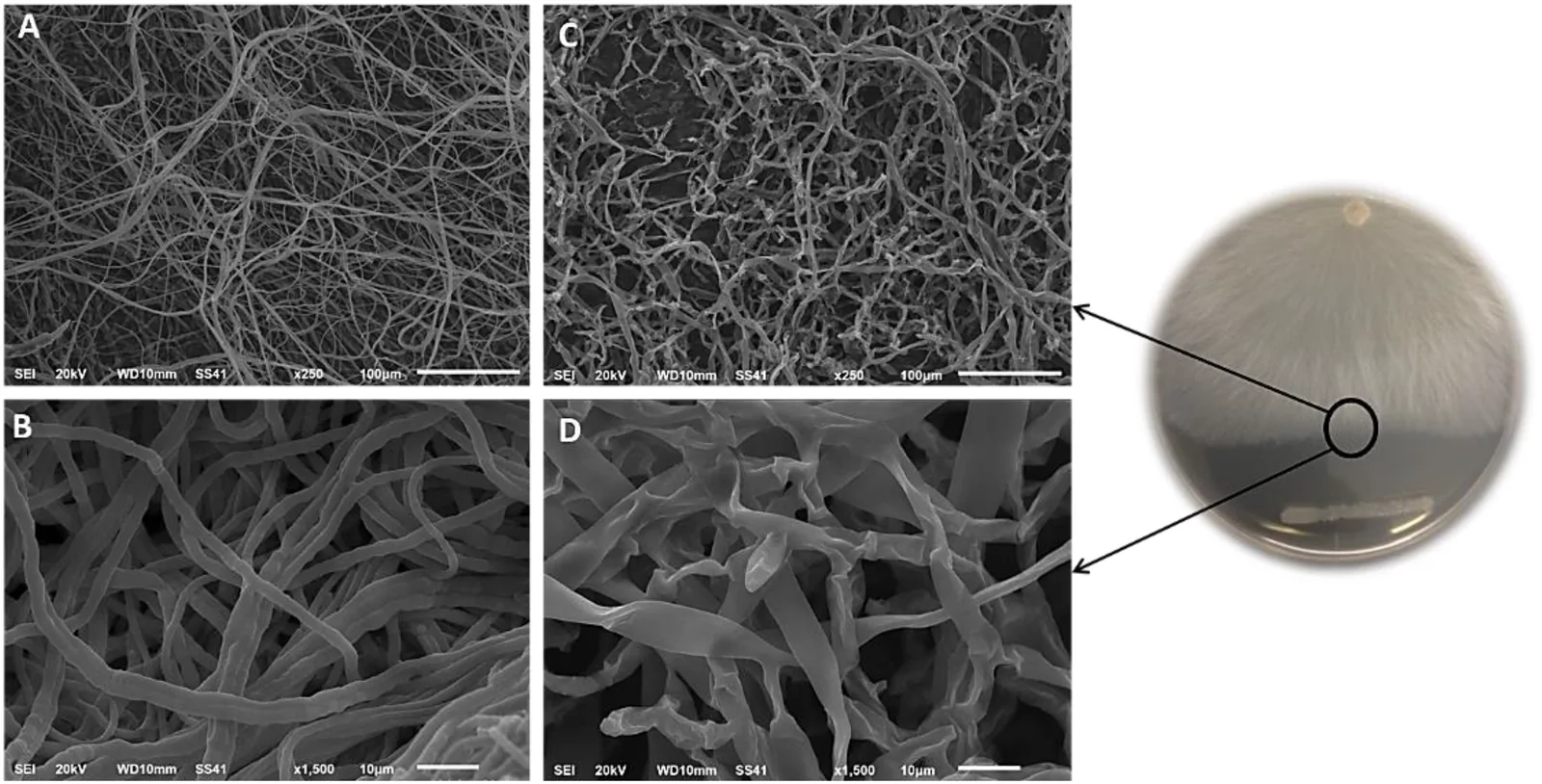
Scanning electron microscopy (SEM) of *R. solani*. The growth characters of root rot pathogen in the control **(A, B)**, and dual culture with *Lysinibacillus fusiformis* **(C, D)**.

SEM analysis revealed that the control *R. solani* had full fungal growth and normal morphological features (**Figures 2A, B**). In contrast, morphological abnormalities, including fungal mycelial lysis, were caused by *L. fusiformis* isolate (**Figures 2C, D**).

### 3.3 Effect of *L. fusiformis* on the incidence of *R. solani* under greenhouse conditions

**Figure 3:**
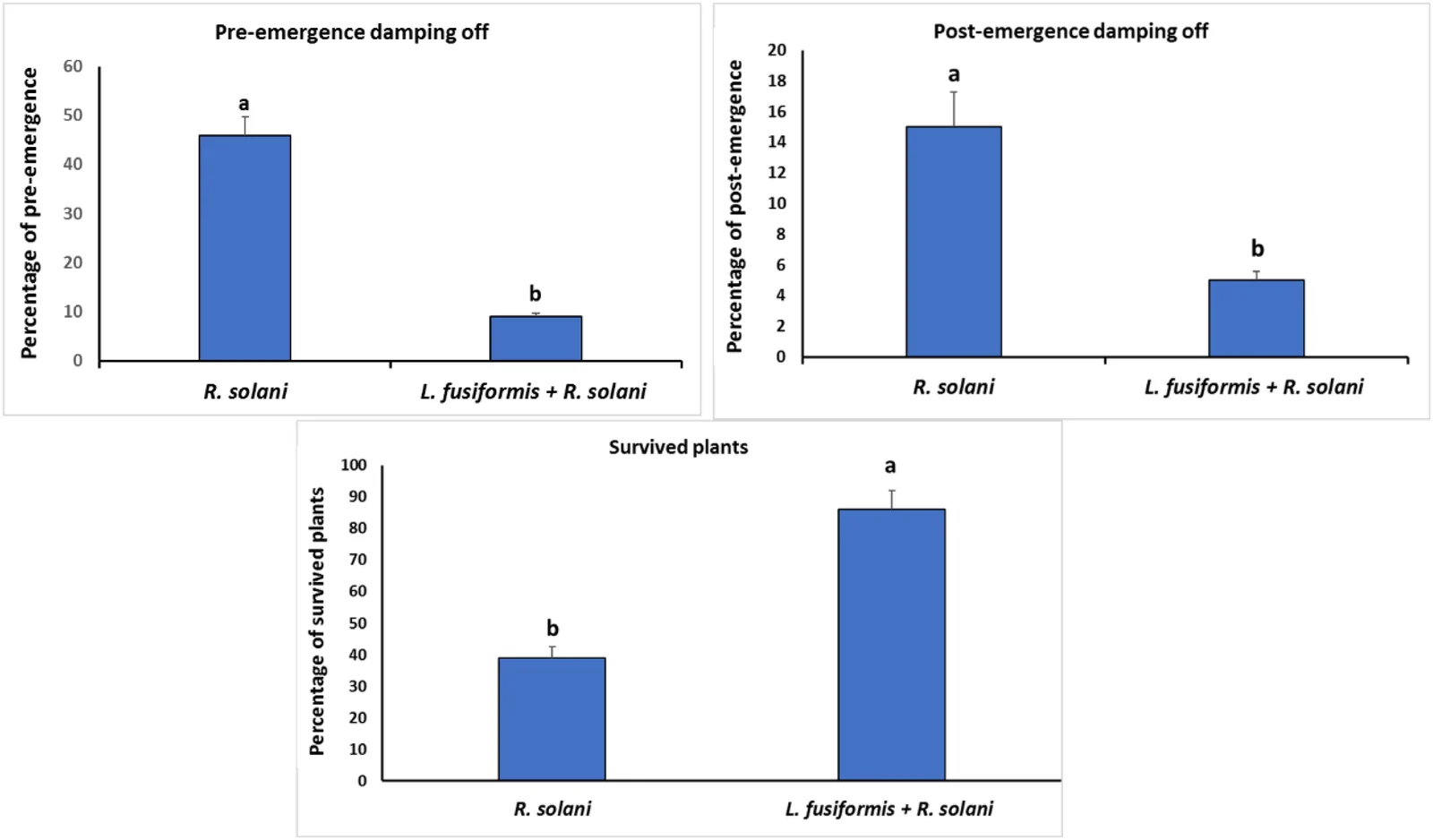
Effect of *Lysinibacillus fusiformis* seed treatment on the percentage of soybean damping-off under greenhouse conditions. Different letters indicate statistically significant differences between treatments.

**Figure 4:**
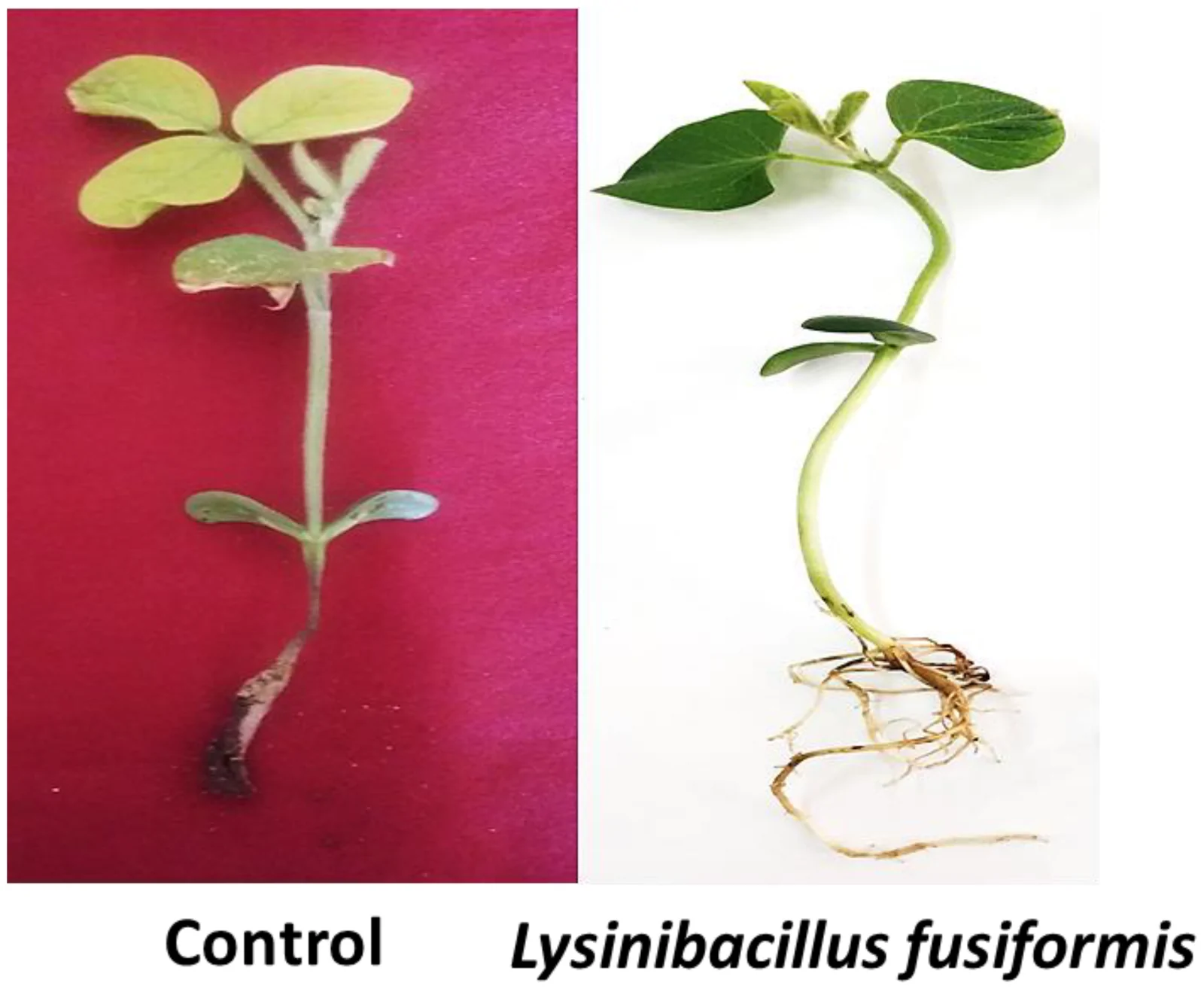
Soybean root rot disease symptoms development caused by *R. solani*. Control group refers to *R. solani* infected plants and the *Lysinibacillus fusiformis* group was seed dressing treatment.

*L. fusiformis* treatment effectively decreased the pre- and post-emergence damping-off percentages with a considerable increase of the plant survival compared to the control grown in soil infected with *R. solani* (**Figure 3**). *L. fusiformis* had greatly decreased the pathogenesis of *R. solani* on the germination of soybean seeds (**Figure 3**). Such effect was also shown in post-emergence records and in the outcomes of plant survival (86%) (**Figure 4**).

### 3.4 Assessment of growth promotion effects

The interaction between plants and microbes in the rhizospheric zone serves to both stimulate plant development and protect it against harmful pests. Plants subjected to *L. fusiformis* outperformed other treatments in terms of shoot length, as well as the number of healthy plants (**Table 2**). Additional evidence of plant growth enhancement was seen in plants that were treated with *L. fusiformis* and then challenged with fungal pathogen *L. fusiformis* increased dry weight of both shoots (3.5 g) and roots (1 g). Soybean plants treated with *L. fusiformis* had the greatest total chlorophyll content since they were the healthiest of all the treatments. Soybean plants, challenged with *R. solani* infection, showed a considerable decrease in chlorophyll content (**Table 2**).

**Table 2:** Effect of *Lysinibacillus fusiformis* on soybean growth parameters under greenhouse conditions.

Treatment*	Shoot length (cm)	Dry weight (g)		Total chlorophyll (mg/g fresh weight of leaf)
		Shoot	Root	
T1	34.6 ± 2.1c	1.3 ± 0.2d	0.4 ± 0.05c	1.2 ± 0.1c
T2	48.7 ± 2.9 b	3.5 ± 0.2b	1.0 ± 0.04b	3.7 ± 0.2b
T3	59.8 ± 2.4 a	4.2 ± 0.3a	1.3 ± 0.04a	4.6 ± 0.2a
T4	46.4 ± 3.1 b	3.1 ± 0.1c	0.9 ± 0.02b	3.9 ± 0.2b

*T1, *R. solani* challenged plants; T2, *L. fusiformis*-treated and challenged with *R. solani*; T3, *L. fusiformis*-treated plants; T4, healthy plants. Different letters indicate statistically significant differences between treatments.

### 3.5 Assessment of defense enzyme activities

The impact of *L. fusiformis* on the plant’s enzymatic system exhibited considerable favorable outcomes (**Table 3**). Enzymatic activity, defined as the quantification of active enzymes, was increased in soybean parts (leaves and roots) treated with *L. fusiformis* and infected with fungal phytopathogen. An incremental rise in enzymatic activity was seen at 10-day-old seedlings. The enzymatic activity of all defense-related enzymes in this investigation was similarly affected. LOX, POD, PAL, and PPO activities were incrementally elevated in *L. fusiformis* treated plants. Comparative analyses of enzyme activity have been conducted across several plant tissues, leaves and roots, under diverse treatments, revealing that the greatest activity was seen in the root tissues. *L. fusiformis* augmented enzymatic activity and stimulated plant defense, as elevated activity was seen in the root and leaf tissues of *L. fusiformis* treated and challenged with *R. solani* compared to *L. fusiformis* treated alone and *R. solani*-infected control (**Table 3**). Soybean plants treated with *L. fusiformis* alone exhibited substantially elevated enzymatic activity relative to the untreated control. The control plants exhibited the minimal enzymatic activity. A comparison of all the defense-related enzymes reveals that POD had the greatest level of expression, followed by PPO, while PAL had the lowest.

**Table 3:** Effect of *Lysinibacillus fusiformis* on antioxidant enzymes (µmol product min^-1^ mg protein^-1^) and total phenolic content (mg/g fresh weight) of soybean plants under different treatments on the 10-day-old seedlings.

Treatment*	POD		PPO		PAL		LOX		Total phenol	
	Leaf	Root	Leaf	Root	Leaf	Root	Leaf	Root	Leaf	Root
T1	2.8 ± 0.2b	6.8 ± 0.4c	2.8 ± 0.2b	5.4 ± 0.3c	0.17 ± 0.02c	0.19 ± 0.02c	0.54 ± 0.03b	0.57 ± 0.02c	4.9 ± 0.2c	6.3 ± 0.3c
T2	4.9 ± 0.6a	9.8 ± 0.4a	3.5 ± 0.4a	8.9 ± 0.4a	0.31 ± 0.03a	0.36 ± 0.02a	0.81 ± 0.04a	0.86 ± 0.02a	6.9 ± 0.3a	8.8 ± 0.3a
T3	4.3 ± 0.7a	8.6 ± 0.3b	3.2 ± 0.4a	7.8 ± 0.3b	0.25 ± 0.03b	0.28 ± 0.03b	0.79 ± 0.03a	0.81 ± 0.02b	5.8 ± 0.2b	7.4 ± 0.4b
T4	1.9 ± 0.2c	4.1 ± 0.2d	1.9 ± 0.2c	3.6 ± 0.3d	0.09 ± 0.01d	0.09 ± 0.01d	0.41 ± 0.02c	0.43 ± 0.01d	3.1 ± 0.1d	4.2 ± 0.2d

*T1, *R. solani* challenged plants; T2, *L. fusiformis*-treated and challenged with *R. solani*; T3, *L. fusiformis*-treated plants; T4, healthy plants. Different letters indicate statistically significant differences between treatments.

Higher phenolic buildup was identified in treated soybean roots challenged with fungal infections. The leaves also showed similar outcomes, but to a lesser extent than its roots (**Table 3**). The plants treated with *L. fusiformis* had phenol concentrations that were lower than those of the challenge-inoculated plants but still greater than those of the control, untreated plants.

### 3.6 Expression analysis of defense-related genes in soybean plants

**Figure 5:**
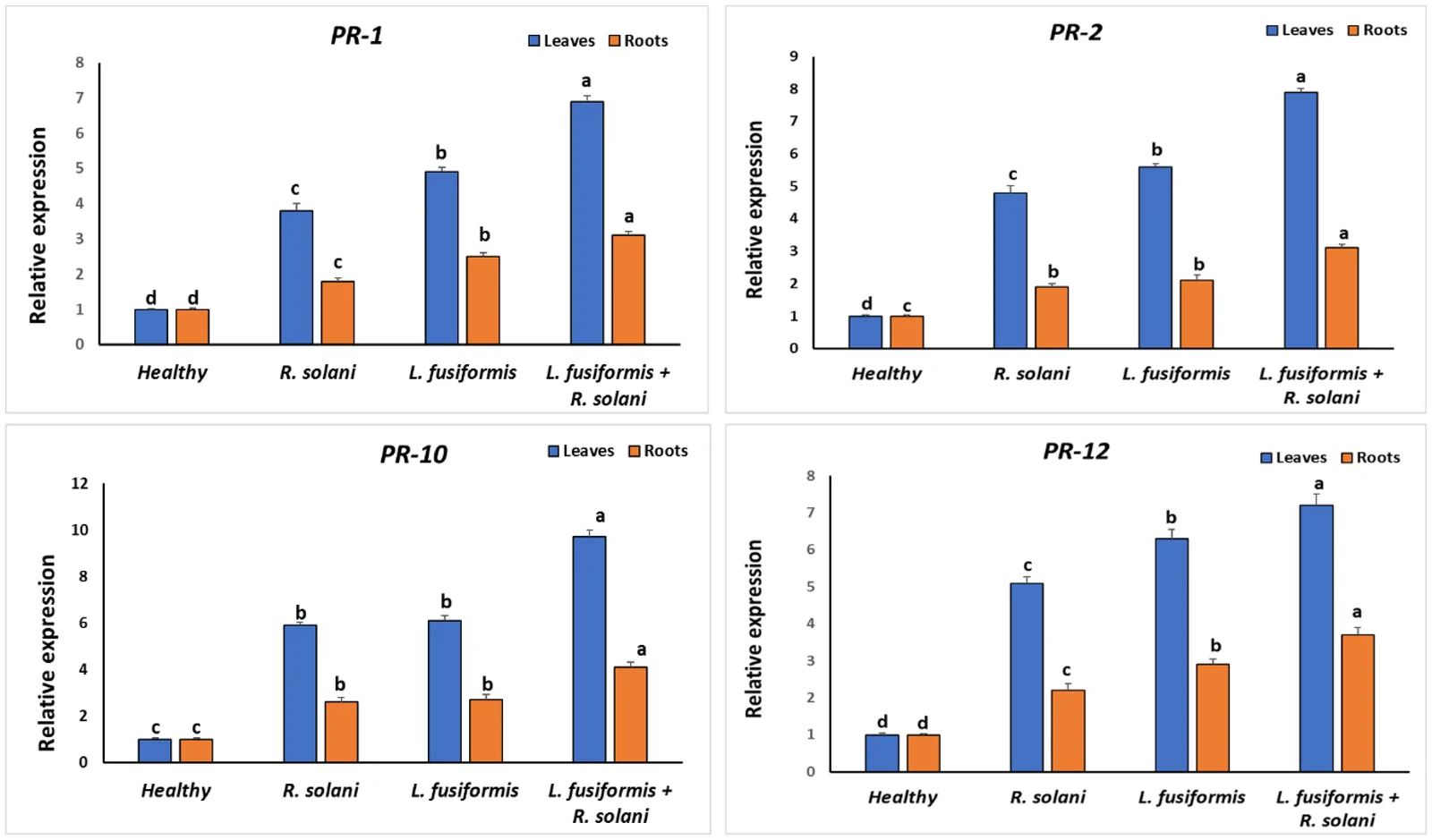
Relative expression of pathogenesis-related genes (*PR-1*, *PR-2*, *PR-10* and *PR-12*) in healthy plants, *R. solani*-infected plants, seed dressing with *Lysinibacillus fusiformis* plants, and seed dressing with *L. fusiformis* and challenged with *R. solani*. Different letters indicate statistically significant differences between treatments.

The results demonstrated that the rhizobacterial inoculation considerably raised transcripts of the most examined genes, with the extent to which this increase was dependent on the presence of *R. solani* (**Figure 5**). Inoculation with rhizobacteria elicited distinct gene responses. The levels of *PR-1, PR-2, PR-10*, and *PR-12* transcriptions were the greatest in the *L. fusiformis* treated and *R. solani* challenged groups in both leaves and roots. However, in both roots and leaves, the levels of *PR-1* and *PR-12* transcripts were significantly higher in stimulated treatment. Remarkably, *L. fusiformis* increased the transcripts of the *PR-10* gene with *R. solani* infection (**Figure 5**).

### 3.7 Effect of *L. fusiformis* on the incidence of *R. solani* under field conditions

**Figure 6:**
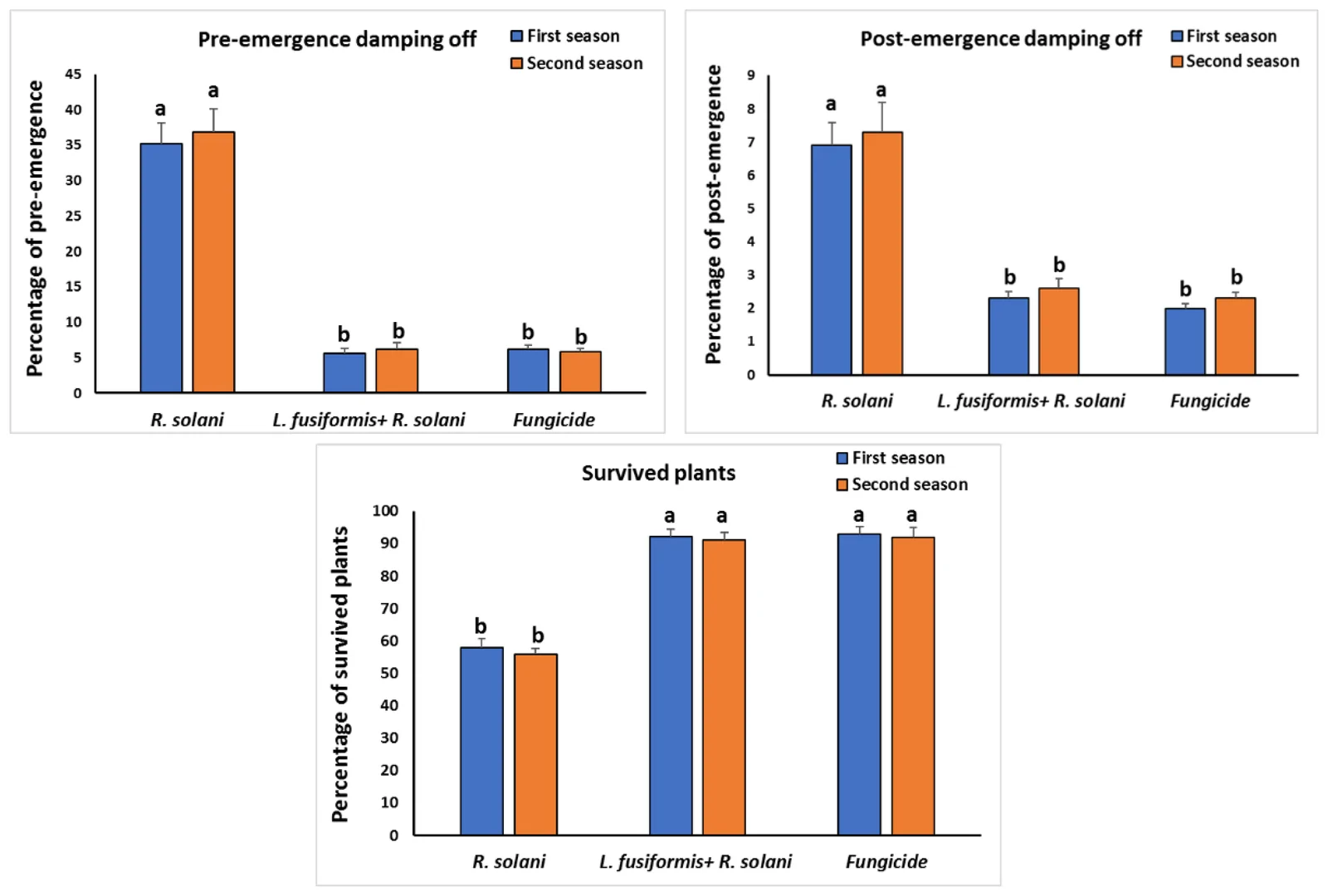
Effect of *Lysinibacillus fusiformis* (as seed treatment) on the percentage of soybean damping-off under field conditions. Different letters indicate statistically significant differences between treatments.

The proportions of pre- (5.6 and 6.2%) and post-emergence damping-off (2.3 and 2.6% for first and second seasons, respectively) were considerably lowered by *L. fusiformis*, as compared to the untreated control at both seasons (**Figure 6**). High disease control values and increased percentage of plant survival, comparable to those obtained with the fungicide treatment, were achieved with *L. fusiformis* treatment (**Figure 6**).

### 3.8 Effect of *L. fusiformis* on yield parameters of soybean plants under field conditions

*L. fusiformis* considerably increased yield parameters of the soybean crop under field circumstances. Significant influence was noticed using *L. fusiformis* (92.8 and 94.0 cm) and fungicide (93.2 and 92.9 cm) treatments on plant height in comparison with the control (65.4 and 62.8 cm for the 2024 and 2025 seasons, respectively). *L. fusiformis* treatment resulted in a considerable increase in the number of soybean branches (4.2 and 4.4) compared to the untreated control (2.5 and 2.3 for the 2024 and 2025 seasons, respectively). There was no discernible difference between *L. fusiformis* treatment (79.5 and 80.1) and the treatment with Thiophanate-Methyl (77.9 and 77.2 for the first and second seasons, respectively) in the case of the number of pods per soybean plant (**Tables 4**, **5**). *L. fusiformis* resulted in a considerable increase in seed weight per soybean plant (33.8 and 34.2 g) as compared to the control group (22.9 and 22.1 g for the first and second seasons, respectively). The weight of one hundred seeds was substantially increased by *L. fusiformis* treatment (19.2 and 19.7 g) in comparison to the untreated control (14.8 and 14.2 g (for the first and second seasons, respectively)). Additionally, *L. fusiformis* and the fungicide treatments were responsible for the greatest increase in seed yield. No significant differences were seen between *L. fusiformis* treatment and the treatment with Thiophanate-Methyl in both seasons (**Tables 4**, **5**).

**Table 4:** Effect of *Lysinibacillus fusiformis* (as seed treatment) on growth and yield measures of soybean under field conditions in the first season.

Treatment	Plant height (cm)	No. of branches plant^-1^	No. of pods plant^-1^	Seed weight (g) plant^-1^	100 seed weight (g)	Seed yield (ton hectare^-1^)
Control	65.4 ± 2.1 b*	2.5± 0.3 b	37.4± 1.8 b	22.9 ± 1.2 b	14.8± 1.1 b	0.38 ± 0.03 b
*L. fusiformis*	92.8 ± 2.5 a	4.2± 0.5 a	79.5± 3.1 a	33.8 ± 1.5 a	19.2± 1.3 a	0.69 ± 0.05 a
Thiophanate-Methyl	93.2 ± 1.9 a	4.1± 0.4 a	77.9± 3.7 a	33.1 ± 1.3 a	18.7 ± 1.0 a	0.67 ± 0.04 a

*Different letters indicate statistically significant differences between treatments.

**Table 5:** Effect of *Lysinibacillus fusiformis* (as seed treatment) on growth and yield measures of soybean under field conditions in the second season.

Treatment	Plant height	No. of branches plant^-1^	No. of pods plant^-1^	Seed weight (g) plant^-1^	100 seed weight (g)	Seed yield (ton hectare^-1^)
Control	62.8 ± 2.9 b*	2.3 ± 0.4 b	34.9 ± 1.6 b	22.1 ± 1.1 b	14.2± 1.3 b	0.35± 0.04 b
*L. fusiformis*	94.0 ± 1.7 a	4.4 ± 0.7 a	80.1 ± 3.9 a	34.2± 1.9 a	19.7± 1.2 a	0.70± 0.06 a
Thiophanate-Methyl	92.9 ± 1.8 a	3.9 ± 0.6 a	77.2 ± 3.5 a	33.3± 1.7 a	19.2± 0.9 a	0.67± 0.05 a

*Different letters indicate statistically significant differences between treatments.

## 4 Discussion

Root rot caused by *R. solani* is becoming an international threat requiring urgent care. Antimicrobial resistance, environmental contamination, and application restrictions in organic farming have all reduced the efficacy of chemical fungicides used to combat this disease (Bernard et al., 2012). Plants often resist the majority of phytopathogens because their immune systems are both effective and complicated, allowing them to manage the vast majority of microbes that populate the environment. The inhibitions of microbial growth by PGPR-mediated ISR may control several plant diseases, which have a substantial impact on plant health (Doornbos et al., 2011). Protecting plants against pathogen invasion is possible by activating their defense systems with the help of a biological inducer. PGPR could provide a sustainable biocontrol method against *R. solani*. However, effective field application is challenging. A bacterial strain that is effective against one group of *R. solani* could be ineffective against another. Therefore, it is essential to match the right bacterial strain with the appropriate pathogen strain (Aydın, 2022). The findings obtained in controlled environments, such as labs or greenhouses, rarely reflect field application. The bacterial resilience and resistance to *R. solani* may be compromised by environmental variables such as fluctuating soil moisture, temperature increases, pH levels, and competition with native soil microorganisms (Liu et al., 2018).

This study used one PGPR strain isolated from the rhizosphere of soybean to control *Rhizoctonia* root rot. Antagonistic bacteria are regarded as optimal biological control agents against phytopathogens due to their rapid growth, ease of handling, and active colonization of the rhizosphere. Research investigations indicate that the application of PGPR as biocontrol agents against soil-borne fungal diseases represents the most effective alternative or integrated method to physical and chemical control (Saraf et al., 2014). Research on the biocontrol mechanisms of *L. fusiformis* in soybean against *R. solani* is anticipated to be improved by this work. The findings demonstrated significant antifungal activity of *L. fusiformis* against fungal infection, effectively inhibiting the development of *R. solani* on dual plate cultures. *L. fusiformis* inhibited *R. solani* growth in soybean. This study explores soybean resistance to *R. solani* by *L. fusiformis*. *L. fusiformis* and Thiophanate-Methyl significantly reduced pre- and post-emergence damping-off as well as increased soybean growth growth and yield components under greenhouse and field conditions. *Pseudomonas* and *Bacillus* were extensively investigated and demonstrated to be very efficient against wilt pathogens, as well as contributing to enhanced crop yield (Gul et al., 2013). Various research findings indicate that *Bacillus* sp. BPR7 significantly suppressed the *in vitro* development of many phytopathogens, including *Macrophomina phaseolina, Sclerotinia sclerotiorum, F. oxysporum, Colletotrichum* sp.*, F. solani*, and *R. solani* (Kumar et al., 2012).

Seed bacterization stimulated soybean to produce defense-related compounds, as shown by higher phenolic contents and defense-related enzymes in plants treated with *L. fusiformis* prior to challenge inoculation. However, the greatest accumulation of defense-related compounds was noted when plants treated with *L. fusiformis* were exposed to the pathogen. The growth of many fungal pathogens was found to be inhibited by the release of antifungal substances (Hassan et al., 2014; Elsharkawy and El-Khateeb, 2019). Treatment of *B. thuringiensis* and *P. flurescence* pf1 in tomatoes has been shown to accumulate phenolics that are effective against soil-borne fungi (Akram et al., 2013). *B. altitudinis* JSCX-1 increases reactive oxygen and callose buildup in soybean, which effectively reduces the incidence of *Phytophthora sojae* (Lu et al., 2017). *In vitro* investigation has shown that exposure of certain phytopathogenic fungi to particular fungal hydrolyzing enzymes, such as chitinases, cellulases, proteases, or glucanases, may effectively degrade the majority of fungal cell walls (Compant et al., 2005). Chitinase synthesized by *B. thuringiensis* GS1 impeded the proliferation of *R. solani.* Although reactive oxygen species (ROS) may promote the host’s immune response to disease, they begin to cause damage to host cells once they exceed a certain level. Antioxidant enzymes including superoxide dismutase (SOD), peroxidase (POD), and catalase (CAT), assist plants in removing ROS (Elsharkawy and Alzahrani, 2026).

Plant growth characteristics, including shoot length and shoot and root dry weight, serve as indicators of plant health and are used for comparative analysis across treatments. In the current investigation, plants treated with *L. fusiformis* showed a notable improvement in the plant growth indices under greenhouse and field conditions. *B. subtilis* CKT 1 has been identified as an effective biocontrol and plant growth-promoting bacterium (PGPB) against *S. sclerotiorum, F. oxysporum*, and *R. solani*, exhibiting various plant growth-promoting properties (Walia et al., 2013). The primary goal of PGPR is to capture atmospheric nitrogen and dissolve insoluble soil phosphate. Additionally, they produce siderophores, which extract iron from the soil and transport it to plant cells. PGPR also synthesizes various plant growth phytohormones, including cytokinins, auxins, and gibberellins, which boost plant growth. Lastly, they synthesize the enzyme ACC deaminase, which reduces ethylene concentrations found in plants. Through an indirect mechanism, PGPR promoted plant development by secreting several cell wall-degrading enzymes that were effective against phytopathogens and by inducing systemic resistance (Elsharkawy and Alzahrani, 2026). The effect of bacteria in enhancing plant development under both normal and stressed situations has been extensively studied (Heidari and Golpayegani, 2012). *Carnobacterium* sp. SJ-5 showed strong Pi-solubilizing, IAA-producing, and ACC deaminase-producing activity (Jain et al., 2014).

qRT-PCR results exhibited increased transcription of defense-associated genes in soybean plants treated with *L. fusiformis* relative to the control. Phytoalexins, β-1, 3-glucanases, chitinases, pathogenesis-related proteins, phenolics, and callose are among the compounds associated with plant defense (Arfaoui et al., 2007; Song et al., 2010). After pathogen inoculation, many genes in legumes that encode proteins involved in pathogenesis are up-regulated both locally and systemically (Robert and Martha, 2010). In addition, it has been shown that some defense genes become more active in response to bacteria which contributes to protecting plants against fungal diseases (Arfaoui et al., 2007). For instance, applying rhizobium has the potential to decrease disease severity by priming plants to respond to fungal infections through the induction of PR genes that are responsible for phytoalexin production (Arfaoui et al., 2007). It is possible that PAL regulates the levels of phenolics in rhizobium-treated seedlings that protect them against pathogen infection (Mishra et al., 2006). Based on our findings, leaf expressions of chosen PR genes were greatly increased by inoculation with *L. fusiformis*. The results showed that the inoculation of soybean plants with *L. fusiformis* activated their defense mechanisms, both locally and systemically. *PR-1* activates whole-plant immunity by releasing a C‐terminal CAPE1 peptide, which is a protein fragment. It inhibits the growth of fungal spores (Han et al., 2023). It attacks the invasive fungal cell walls. Long sugar chains constitute the cell walls of fungi. *PR-2* disrupts these chains, resulting in the necrosis and death of the invader’s cells (Kumar et al., 2025). *PR-10* has the ability to degrade the genetic material of invading fungi. Additionally, it facilitates the coordination of responses to plant stress (Dos Santos and Franco, 2023). *PR-12* causes damage to the membranes of fungi. It punctures the invaders, resulting in the loss of essential nutrients (Dos Santos and Franco, 2023).

## 5 Conclusion

Soybeans treated with *L. fusiformis* as a seed dressing showed a decrease in pre- and post-emergence damping-off under greenhouse and field conditions. The total chlorophyll content, antioxidant enzyme activities, and shoot and root growth of soybean were all significantly enhanced by treatment with *L. fusiformis*. Additionally, PR gene transcriptions were stimulated in *L. fusiformis*-treated soybean plants. The application of *L. fusiformis* may improve soybean growth and defense against *R. solani*. The data gathered might be a useful resource for further studies. This research is the first evaluation of *L. fusiformis* against soybean *Rhizoctonia* root rot under Egyptian field conditions.

## Data Availability

The raw data supporting the conclusions of this article will be made available by the authors, without undue reservation.
